# Rule-based spatial modeling with diffusing, geometrically constrained molecules

**DOI:** 10.1186/1471-2105-11-307

**Published:** 2010-06-07

**Authors:** Gerd Gruenert, Bashar Ibrahim, Thorsten Lenser, Maiko Lohel, Thomas Hinze, Peter Dittrich

**Affiliations:** 1 Friedrich Schiller University Jena, Bio Systems Analysis Group, Ernst-Abbe-Platz 1-4, 07743 Jena, Germany; 2 German Cancer Research Center, Im Neuenheimer Feld 581, 69120 Heidelberg, Germany; 3 Jena Centre for Bioinformatics (JCB), Jena, Germany; 4 Friedrich Schiller University Jena, School of Biology and Pharmacy, Department of Bioinformatics, Ernst-Abbe-Platz 1-4, 07743 Jena, Germany

## Abstract

**Background:**

We suggest a new type of modeling approach for the coarse grained, particle-based spatial simulation of combinatorially complex chemical reaction systems. In our approach molecules possess a location in the reactor as well as an orientation and geometry, while the reactions are carried out according to a list of implicitly specified reaction rules. Because the reaction rules can contain patterns for molecules, a combinatorially complex or even infinitely sized reaction network can be defined.

For our implementation (based on LAMMPS), we have chosen an already existing formalism (BioNetGen) for the implicit specification of the reaction network. This compatibility allows to import existing models easily, i.e., only additional geometry data files have to be provided.

**Results:**

Our simulations show that the obtained dynamics can be fundamentally different from those simulations that use classical reaction-diffusion approaches like Partial Differential Equations or Gillespie-type spatial stochastic simulation. We show, for example, that the combination of combinatorial complexity and geometric effects leads to the emergence of complex self-assemblies and transportation phenomena happening faster than diffusion (using a model of molecular walkers on microtubules). When the mentioned classical simulation approaches are applied, these aspects of modeled systems cannot be observed without very special treatment. Further more, we show that the geometric information can even change the organizational structure of the reaction system. That is, a set of chemical species that can in principle form a stationary state in a Differential Equation formalism, is potentially unstable when geometry is considered, and vice versa.

**Conclusions:**

We conclude that our approach provides a new general framework filling a gap in between approaches with no or rigid spatial representation like Partial Differential Equations and specialized coarse-grained spatial simulation systems like those for DNA or virus capsid self-assembly.

## Background

Modeling and simulation of biochemical networks as well as the integration of experimental data provide powerful tools to gain insight into the complexity of living systems [1]. Possibly even more important and seen as the next step is the transition to a predictive biology [2-4], which has been accomplished in physics long ago [5]. But many biochemical networks are hard to treat and describe explicitly. They are too complicated to be overseen just by listing all the reactions. This happens especially fast when effects of combinatorial complexity are involved, for example, in cases of post-translational modification of multiple sites on a protein or large multi-subunit complexes [6-9]. A new species would then have to be used for every state or combination of the molecules. Unfortunately, this complexity seems to be an integral part in living systems. Most important cellular functions like ATP synthesis or transcription involve the cooperation of multiple proteins forming complexes [10,11]. Examples are the death-inducing signaling complex (DISC) [6,12], the epidermal growth factor receptor (EGFR) [13] with 9 phosphorylation sites or the tumor suppressor protein p53 with 27 phosphorylation sites. The latter one could theoretically assume up to 2^27 ^= 134, 217, 728 different phosphorylation states [14]. Microtubules [15], viral shells [16,17] or hybridizing DNA strands [18] constitute additional examples for structures formed by complex interactions. To cope with this combinatorial complexity, reactions systems have to be defined implicitly [6,19], i.e., by using implicit reaction rules operating on molecules possessing a structure.

### Rule-Based Modeling

Rule-based modeling approaches share the idea of subdividing molecules into their *components*, denoting protein domains, active sites or any other feature of the particle [6,9]. These sites can then be modified post-translationally or bind to subdomains of other molecules. This concept is referred to as the *domain-oriented approach *[20,21]. Among others, available software packages for rule-based modeling are Moleculizer [22], Stochsim [23], BioNetGen [24], Pathway Logic Assistant [25], BIOCHAM [26] or Cellucidate.

**Figure 1:**
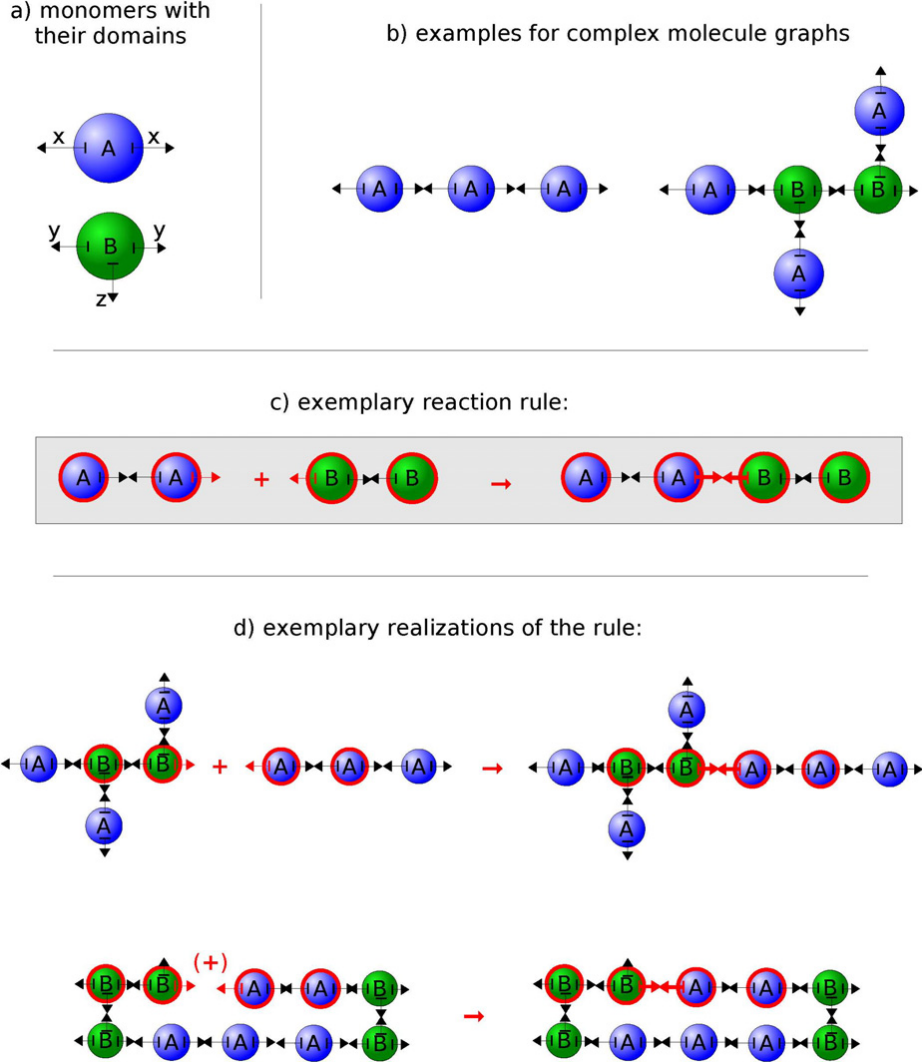
**Exemplary rule-based system**: Two elementary molecule types (blue, green) with their subdomains (or *components*) are displayed (a). Each component can be bound to another component or can be modified, e.g. denoting a phosphorylation or a conformational change. Site names need not be unique and hence a wide spectrum of possibilities for the system's specification is offered. Multiple elementary molecules can be connected at their components to form complex *molecule graphs *(b). Reaction rules, for example the *binding reaction *(c), are specified by using patterns graphs (or *reactant patterns*). A reactant pattern fits to a molecule graph, if it is contained as a subgraph in the molecule graph. Note that some components are missing in the reactant pattern's definition, which are then ignored in the matching process. Two different instances of the reaction rule are symbolized (d). In the upper realization, two independent molecule graphs are connected. For the lower example on the other hand, both of the rules' reactant patterns are found in a single connected molecule graph.

In this paper, we assume that a complex molecule is described by a *molecule graph*, which consists of *elementary molecules *that are connected with each other. Each elementary molecule can further possess a set of subdomains called *components *(see Figure 1, a). Subdomains serve either as connectors between elementary molecules or can be modified, e.g., phosphorylated.

The set of all possible reactions is implicitly defined as the rule-based reaction system (*R, P, S*) with the set of rules *R*. *R *is based on the assumption, that there are groups of chemical species *s *∈ *S*, sharing a common property (or pattern) *p *∈ *P*. Hence they can be subject to a related set of reactions, summarized by a reaction rule *r *∈ *R*. In our case, the common property might be the containment of a similar subgraph structure, for example. Each pattern *p *defines an equivalence class of species from *S *by the function *Eq*_*S*_(*p*) ⊆ *S*.

For a start, we return to a non rule-based reaction system (*R*', *S*). For a simpler description, we will only consider bimolecular reactions. Each reaction *r*' ∈ *R*' would then consist of a quadruple of molecular species *s *∈ *S*.

An instance of this reaction *r*' happening in the simulation of the reactor would then consume one molecule of the species *s*_1 _and one molecule of the species *s*_2_. On the other hand, it would produce one molecule of the species *s*_3 _and one molecule of the species *s*_4_. The species participating in the reactions and the process of exchanging the molecules are defined unambiguously. To define *reaction rules *instead of the reactions themselves, we only have to exchange the set of species *S *with the set of patterns *P*, giving:

An instance of the reaction rule *r *happening in the simulation of the reactor would consume one molecule of a species from the equivalence group *Eq*_*S*_(*p*_1_) and one molecule of *Eq*_*S*_(*p*_2_). In exchange, it would produce one molecule of a species from *Eq*_*S*_(*p*_3_) and one molecule of *Eq*_*S*_(*p*_4_). Now the product site of the reaction rule is not specified unambiguously. The exact species to be produced has to be derived from the actually consumed species and from the type of reaction rule.

Non rule-based reaction systems typically allow only the one type of reaction that consumes one set of molecules and/or creates another. These are called *exchanging rules *in our approach. In contrast, there are more delicate types of rules conceivable for rule-based systems. One typically starts with the assumption that molecules do not usually vanish or emerge. Instead, their components are connected, disconnected or modified. Hence the most important rules for our approach form or break bonds between molecules' components or modify them (see Figure 1). These rule types are called *modifying-*, *binding- *and *breaking rules*.

Different rule types that are not yet supported in our simulator might for example exchange subsets of molecule-graphs specifically or change some molecule's conformations.

### Spatial Modeling

Spatio-temporal heterogeneous distributions of biomolecules have an important impact on the function of biochemical systems [7,27-32].

For that reason, a variety of spatial simulation techniques for reaction networks was developed ranging from macroscopic systems like (Stochastic) Partial Differential Equation [33] to Brownian Dynamics [34] and Molecular Dynamics [35] approaches at the micro scale. A rich set of software packages is available for the spatial simulation of explicitly defined reaction networks. Examples are MCell [36], Cell++ [37], Virtual Cell [38,39], SmartCell [40], mesoRD [41], Smoldyn [42], Project Cybercell [43] or ChemCell [44]. While our notion of "space" in this paper focuses on the particle and macromolecule geometry, also the geometric aspects of the reactor might be of interest [44,45].

But the combination of spatial and rule-based representations has only been addressed by few approaches. An interesting development is StochSim [23] that can operate on a two-dimensional lattice and offers multistate molecules but no multimerizations. Another system, the event-based simulator for spatial assembly problems [46,47] focuses on self-assembly mechanisms. Instead of rules and reactant patterns as described before, it uses "local rules" [48] to assign each type of binding site a set of other site types it can bind to. Nonetheless, there is more potential in the combination of spatially heterogeneous concentrations, spatially structured molecules and rule-based modeling than covered by these methods. The spatial features of the molecules, in particular the volume exclusion, their geometrically constrained interactions and hence also their ability to form three dimensional structures, might severely influence various further effects in a combinatorially complex reaction network. Examples are molecular crowding [49], orientation dependent reaction probabilities and steric effects [50,51], various polymerizations and self-assembly processes [15-17] including hierarchical assembly pathways [47] or the function of molecular machines [10,11].

In the next section we will present our approach for rule-based modeling in space and describe our actual implementation called SRSim. Afterwards, results of our *in silico *studies will be presented, revealing the qualitative assets of the combination of rule-based and spatial modeling. Finally, we discuss the implications for the analysis of complex bio-chemical systems and open issues for rule-based modeling in space.

## Methods

### Rule-Based Modeling in Space

Independent of our own implementation that is described in the next section, we suggest the following general features for a spatial, rule-based reaction system: Similar to the domain-oriented [20,21] and rule-based modeling approaches, a molecule consists of *elementary molecules *(EM), that are compiled to a *complex molecule graph*. Each EM belongs to an elementary species, which we extend by further information, such as size, mass, diffusion coefficients, geometry and orientation of binding sites - dependent on the particular chosen spatial simulation model. Note that we use space in a broader sense than other approaches that utilize Partial Differential Equations [38,39] or spatial variants of the Gillespie algorithm [40,41,52] for the simulation of a heterogeneous distribution throughout the reactor. In what we consider a spatial, rule-based model, a complex molecule should also have a form and volume due to the geometry of its EM. This does not only imply possible geometries for the complex molecule graphs, but also constrains the possible reactions. That is, only those molecules can undergo a reaction that (i) are geometrically compatible and that (ii) fit in the pattern of a reaction rule. In spite of that, the definition of the reaction rules is the same as in "conventional" rule-based modeling approaches. What has to be provided additionally is the geometry of the EM and parameters for the spatial simulation, such as diffusion rates and reactor properties. If the number of complex species is bounded, the set of all possible complex molecule graphs (or complex species) and reactions can be generated in advance. Alternatively, new complex species can be generated just in time, when a reaction occurs that generates this species. The dynamical simulation takes place in Euclidean space, where each complex molecule graph has a location and orientation that is given implicitly (by the locations of its constituting EM) or explicitly (by an own position and orientation vector).

### The Simulation Tool SRSim

We developed an integrated spatial and rule-based simulation software called SRSim. It combines the modeling-strengths of a rule-based software like BioNetGen [24] or Stochsim [23] with a stochastic, diffusing-particle-based simulator like Smoldyn [42] and force-mediated interactions, which are possible in Molecular Dynamics software packages like LAMMPS [53]. The supplied prototypic software is meant as an exemplary and extensible implementation of this modeling approach and certainly has to be adapted for particular problems.

The description of the rule system is extracted from files in the BioNetGen Language (BNGL) [6] to allow an easy im- and export of models from BioNetGen. The description of the reactor properties and the molecular geometries are specified independently. Nonetheless, since the reaction system operates on pattern subgraphs, there is no need to generate all possible complex species or reactions in advance, which saves computational ressources. The spatial model aimed at by SRSim is settled in the meso-level, between microscopic all-atom Molecular Dynamics simulations and the macroscopic use of Partial Differential Equations. Similar to a viral shell model used in [16], each complex molecule graph is composed of spheric elementary molecules (EM). The species of the EM then reveals information about the mass, radius, diffusion rate, geometric properties and the set of components that are attached to this EM.

EM move through continuous 3D space, while time is discretized in small steps typical for Molecular Dynamics simulators. An EM's movement is influenced by Brownian Motion using Langevin Dynamics [54] as well as by forces arising from interactions with other EM through bonds and volume-exclusion effects. The positions of complex molecule graphs are not considered explicitly in the spatial simulation but move implicitly with the movement of their constituting elementary molecules' particles.

#### Software Layout

SRSim is realized as extension to the open source Molecular Dynamics simulator LAMMPS [53]. The new set of C++ classes uses a self-contained part for the treatment of the implicit reaction system, called the *Rule System*. The other part is a simulator dependent set of connecting *LAMMPS Modules*. Therefore a possible later adaption of SRSim based on different spatial simulators is already prepared. The sources for the Rule System and the LAMMPS Modules are released under the GPL and are included in the additional file 1 - SRSimSrc.zip. The most recent versions of the simulator will be available on our website http://www.biosystemsanalysis.de.

#### Geometry Model

**Figure 2:**
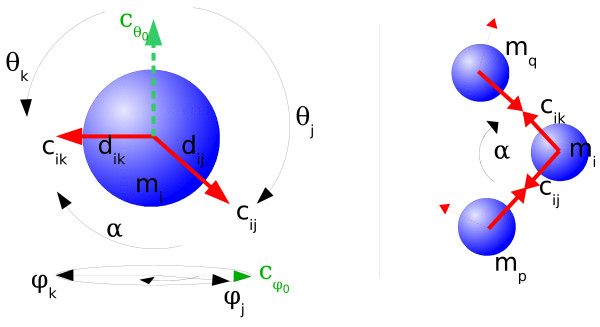
**Exemplary SRSim geometry**: On the left, a molecule *m*_*i *_with two components *c*_*ij *_and *c*_*ik*_, given in polar coordinates, is depicted. The angle *θ *can be imagined as descending from an imaginary polar component , while *φ *rotates the component in the equatorial plane from the imaginary zero-meridian *φ*_0_. The lengths from the center are given with *d*_*ij *_and *d*_*ik*_. The angle *α *that is not specified but calculated from the polar coordinates is the effective angle between the components *j *and *k*. Three molecules and two bonds are shown on the right side. Because the middle molecule is connected with two other molecules (thick red arrows), the angle *α *would be realized under ideal conditions.

From the broad range of possibilities to implement a spatial simulation (see [30,31] for reviews), we chose an individual representation of each elementary molecule in continuous space and discretized time steps, typical for Molecular Dynamics simulations. The location, form and orientation of complex molecule graphs are not described but are given by the positions of their elementary molecules. Similarly it would not be necessary to describe the position and form of a house if the position of every brick was known. The simulation is running in a cuboid box of selectable dimensions and boundary conditions. Even so, more elaborated reactor geometries can be defined through the scripting language of the molecular dynamics simulator. Every *elementary molecule *(EM) *m*_*i *_of the elementary species *M*_*i *_is represented by a single sphere with a given mass *g*_*i*_, radius *r*_*i *_and position *x*_*i*_. Each *component c*_*ij *_of an EM *m*_*i *_can be imagined as a vector starting from the center of the sphere *x*_*i*_. It is given in polar coordinates, by a distance *d*_*ij *_and two angles *θ*_*ij*_, *φ*_*ij *_(see Figure 2, left). *Bonds *between two EM are the straight connection of two component vectors *c*_*ij *_and *c*_*kl *_with the length *d*_*ij *_+ *d*_*kl*_. By forming bonds between the components of the EM, complex molecule graphs can be assembled. We plan to introduce the option to use further geometric features like dihedral angles or rotational orientations for the EM as well, at the price of more complex computations and more possibly unknown parameters. Implications of the current detail level are described in the Discussion Section.

The basic cycle of Molecular Dynamics simulations propagates the time by the small time step Δ*t *in the following way: Starting from a time *t *with known positions *x*(*t*), forces *f*(*t*) and velocities *v*(*t*) of all particles, the positions *x*(*t *+ Δ*t*) at the time *t *+ Δ*t *are calculated as a function of *f*(*t*) and *v*(*t*). Then the forces *f*(*t *+ Δ*t*) and velocities *v*(*t *+ Δ*t*) for the next time step are computed.

Several force calculations are employed to sustain the bond radii and bond angles as undamped harmonic springs. So for each connected component, there's a harmonic force term with the potential

added to LAMMPS' force calculation. Where *K*_*d *_is the spring constant and *d *is the current distance between the connected elementary molecules. Imagine the following molecule graph built from three elementary molecules *m*_*i *_*- m*_*j*_*- m*_*k*_. When two or more connections to the molecules *m*_*i*_, *m*_*k *_set out from one central elementary molecule *m*_*j*_, a harmonic angular force term with the potential

is added to LAMMPS' force calculations for each combination of two connected elementary molecules *m*_*i *_and *m*_*k*_. Here, *K*_*α *_is the spring constant, *α *is the current angle between the EM *m*_*i*_, *m*_*j *_and *m*_*k*_. *α*_*ijk *_is the ideal angle calculated from the geometry definition. So the central EM *m*_*j *_with *n *attached EM implies a set of  angular force terms. *K*_*α *_and *K*_*d *_can only be set generally for all bond types together at the moment but will be set per elementary species in the next SRSim version. The ideal bond angles *α*_*ijk *_are calculated by SRSim from the specified components' polar coordinates (see Figure 2, right) in the initialization phase of the simulation.

The minimal distances between two EM are maintained by a soft-sphere potential:

with the distance *r *between two molecules *m*_*i *_and *m*_*j*_, the cutoff distance *r*_*c *_and the maximal potential *A*. (See the LAMMPS documentation for more information). The result of this potential is a repulsion between molecules, once they move closer together than the sum of their radii. To simulate the Brownian movement of a particles *m*_*i*_, Langevin Dynamics [54,55] is employed by adding a term for random *F*^*R *^and a term for viscous *F*^*D *^forces to the systematic forces  of the model.

where  is the friction coefficient, *v*_*i *_is the particle velocity, *k*_*B *_is the Boltzmann constant, *T *is the temperature, *ξ*_*i*_(*t*) is a gaussian random function and *D *is the diffusion coefficient. While the magnitude of the random and viscous forces are correlated by the *fluctuation-dissipation *theorem, the strength of the repulsive, bond and angular forces can be varied independently. The diffusion rate can be adjusted for each EM individually, whereas the diffusion of complex molecule graphs will emerge from the diffusional behavior of its compounds and the bond forces. As the EM cannot pass through each other, larger complexes' volume-exclusion behavior is also accounted for, given that no large free spaces are left between connected EM.

#### Reaction Model and Kinetics

**Figure 3:**
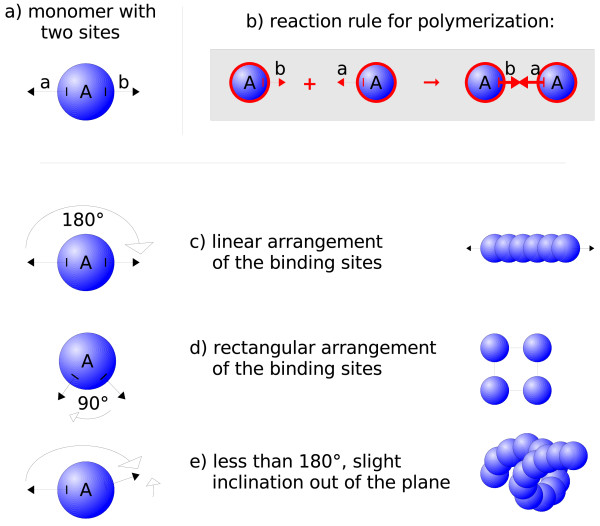
**Dependence of Geometry**: Starting from a molecular species with two binding sites (a), the polymerizations following the simple complexation rule (b) may lead to very different molecule complexes. When assuming a linear conformation of both binding sites (c), linear, rod-like structures will assemble. Using a 90 degree angle between both components (d) would mostly lead to closed quadratic structures. Other geometries including inclinations out of the plane (e) can create helices of different radii and helicities, etc. Please note, that the geometry model that is necessary to distinguish closed quadratic structures and helices from a worm-like chain in (d) and (e) requires the use of dihedral angles (See Section Discussion for details). While dihedral angles are not yet implemented in our software SRSim, similar effects can be achieved by using slightly more complex elementary molecules, as it was done in out spheric self-assembly simulation.

After each update of the particle positions *x*(*t*) in the spatial simulator, the Rule System evaluates the reactor for possible reactions that can happen in the interval [*t, t *+ Δ*t*). Please note that the movement of the particles within this time is not considered for the calculation of reaction probabilities (See the Discussion Section for implications). Our simulator currently supports mono- and bimolecular modifying, binding and breaking reactions (see Section Background for a description of the different rule types). Here, even reactions happening inside of one complex molecule graph may be seen as bimolecular reactions, if different subgraphs of the complex are considered (see Figure 1d). We renounced from implementing exchange rules that would delete one set of reactants while creating another so far. Hence all reactants have to be present in the simulator from the beginning. Sets of new molecules can be added at predefined time steps, but cannot be annihilated yet. This restriction is not an implication of our simulation approach but was simply not needed in our examples so far. It will be implemented in the next version of our software. We do not pre-generate all possible reactions and species but directly apply the reaction rules to the molecules in the reactor that belong to certain reactant patterns. This procedure saves memory and computation time, especially when potentially infinite reaction systems are involved and when the specified geometries constrain the subset of reactions that is actually possible (see Figure 3). Similar to BioNetGen [24] versions later than 2.0, SRSim internally uses a graph-based representation [56] of *reactant patterns *and molecule graphs. Basically, all choices whether and what reactions are to be executed are performed on the set of reactant patterns instead of the actual species. Therefore, all reactant patterns that are present in the rule definitions are enumerated and associated with indices, during the initialization phase. In the running simulation, each elementary molecule stores the indices of the reactant patterns that it currently belongs to.

**Figure 4:**
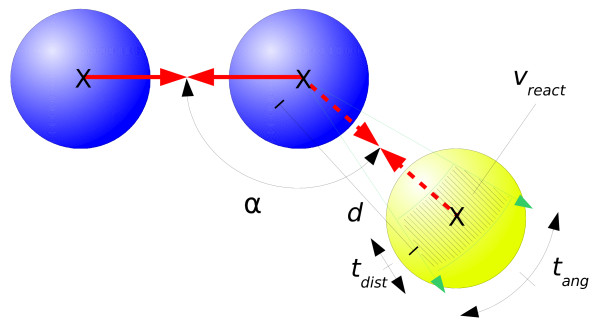
**Creation of Reactive Volumes through Geometric Tolerances**: This graphic is a projection of the three-dimensional system in two dimensions and hence only uses one torsional angle instead of two. Given the molecule graph of the two blue elementary molecules, a reaction with a third, yellow molecule would ideally occur under an angle of *α *and the distance of *d *(the sum of both involved site lengths). But since the exact combination of angles and distances would hardly ever be satisfied in the time-discretized simulation, the bond tolerances *t*_*ang *_and *t*_*dist *_were introduced. They describe a volume *V*_*react *_(or an area in this two dimensional graphic) that can accommodate a possible partner for a reaction. Here, *V*_*react *_is only shown from the blue molecule graph's point of view, while the yellow molecules would create another reactive volume. A reaction between two molecules is only allowed if they both are situated in each other's reactive volumes.

Eventually two neighboring elementary molecules *m*_*i *_and *m*_*j *_can quickly be checked against bimolecular reaction rules that could possibly happen between them, once they are positioned closer together than a threshold *sigma *in the current time step. If their assigned reactant patterns allow the application of at least one reaction rule, both molecules are tested for their "geometric compatibility", which is depending on the values of their *component tolerances*. A reaction may occur only, if the relative positions of *m*_*i *_and *m*_*j *_are deviating less than the given component tolerances *t*_*dist *_and *t*_*ang *_from the ideal bond distance and bond angle. Hence, for each molecule *m*_*i *_there exists a defined *reactive volume * that a possible reaction partner *m*_*j *_can lie in (see Figure 4) and vice versa. This concept is similar to the model of "reactive patches" [27] on spheric molecules or the existence of energy funnels for protein docking [57]. When both molecules are lying in each others reactive volumes, we treat these molecules like existing in a microscopic, non-spatial reactor on their own. The probability of a reaction between *m*_*i *_and *m*_*j *_in the infinitesimal small time interval *dt *is then *k*_2*mic*_*dt*. Considering the current time step of the length Δ*t*, the probability is then exponentially distributed and sampled from *P*(*m*_*i *_reacts with *m*_*j*_) = 1 - [58,59]. See the additional file 2--KineticsAndApplicability.pdf for a discussion on how to convert macroscopic reaction rates to the microscopic reaction rates that are employed here.

When a reaction occurred, the reactant pattern indices for all involved molecule graphs have to be updated. This might sound like a huge computational effort for each reaction, when large polymers or complex molecular networks are considered. The actual work necessary to perform this operation is mostly quite tractable, though: The important value here is the maximum graph diameter *d*_*max *_of all the reactant pattern graphs. There can be no two elementary molecules in one reactant pattern that are more than *d*_*max *_nodes apart. Hence it suffices to recalculate the reactant pattern indices up to a distance of *d*_*max *_from the elementary molecules that were modified by the reaction.

For monomolecular reactions, Gillespie's algorithm [58,60] is operating on the vector of occurrences of the reactant patterns. At the beginning of time step *t*, the time *τ *until the next monomolecular reaction happens is sampled in dependence on the monomolecular reaction propensities . If *τ *is smaller than the time step Δ*t*, an arbitrary molecule that belongs to the reaction's reactant pattern is selected and modified according to the reaction rule. This procedure is repeated until *τ *is greater than the spatial simulation's time step Δ*t*, in which no reaction occurs in this time step. Even if no reaction occurs over several time steps Δ*t*, this fragmented execution of Gillespie's algorithm is equivalent to the original, since the process is "memoryless".

While we calculate the time until first order reactions happen independent from the spatial simulation, the effective macroscopic reaction rates of second order reactions depend on further parameters: To begin with, the *diffusion rate D *influences the movement of the simulated particles and thus determines the collision probability causing an upper bound for the macroscopic reaction rates. We introduced another parameter, the *refractory time*, to solve a problem in the interaction between binding- and breaking rules. After a breaking reaction occurred, two molecules will still be located very close to each other and a secondary binding reaction might reconnect the molecules again quickly in most cases. Thus, to reach a single net dissociation, a series of many successive breaking and binding reactions would have to occur ineffectively. The same problem is encountered in the stochastic spatial simulator Smoldyn [42] and solved by placing both molecules a certain distance apart after cleaving the connection. Because this approach would lead to nonlinear particle movement, which is impractical when the forms and structures of assembling complex molecule graphs are constantly considered, a different solution is used in SRSim. We assign a refractory time to a molecule after one of its bonds is deleted. For this period, the molecule cannot undergo a new binding reaction and has time to move away by diffusion. Please see the additional file 2--KineticsAndApplicability.pdf for a closer analysis of possible influences of the refractory time on the system behavior.

## Results

To demonstrate the emergent effects arising when diffusing, geometrically constrained particles are used in relative simple models, four exemplary applications will be presented. These models were engineered to test and demonstrate our approach, rather than to deliver a highly detailed representation of a special biological system. Consequently, the parameters are kept as simple as possible with arbitrary units. In order to allow rapid experimentation, kinetic parameters, diffusion rates and concentrations are chosen high enough to create results in short simulated times, leading to experiments which can be calculated on a single workstation in computation times of some minutes to hours.

The input files for the presented experiments are included in the additional file 3--ExamplesSrc.zip and short avi movies showing the simulated reactor can be found at http://users.minet.uni-jena.de/~dittrich/tmp/srsim/.

### Scaffold Proteins

Scaffold proteins bind other proteins and are thought to help isolating different signaling pathways but also to catalyze reactions by co-localizing their ligands (For reviews see [61,62]). The following example is not intended to be an exact simulation of the biological process, but to present the potential impact of spatial features on a reaction network model.

**Figure 5:**
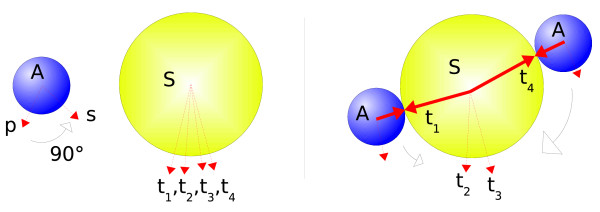
**Simple Scaffold Protein Model**: The system is populated with two types of molecules. The proteins to be phosphorylated *A *and the scaffold proteins *S *are shown in the left panel. All four components of *S *are directed to one of its poles, but since angular tolerances are chosen very high in this example, new bonds are accepted from any angle. When angular forces are turned on, the particles *A *are forced towards one pole of the scaffold (right panel).

Simulations were carried out with two different molecular species. Particles of the first species A can phosphorylate each other's components when they meet in the reactor. Phosphorylations are lost over time. Larger spheric scaffold proteins S can bind up to four particles A with a rate of *k*_*s *_(see Figure 5). While unphosphorylated proteins A stick to the scaffold, phosphorylated particles dissociate quickly. To allow a smaller protein to bind from any direction onto S, we set the angular tolerance to 180°. Since geometrically all the component vectors of S face towards one pole, all bound particles A would be forced to this one pole as well. To facilitate free diffusion for the molecules A on the surface of S, we initially reduce the angular force term to zero.

**Figure 6:**
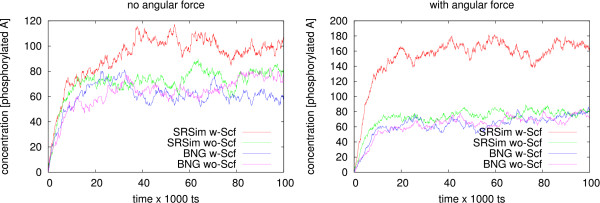
**Effect of Scaffold Proteins**: Each line corresponds to the number of phosphorylated particles of species *A *in a different simulation. Simulation in SRSim and BioNetGen enabled and disabled the binding of molecules *A *to scaffold molecules *S *through a change in the *k*_*s *_rate. "w-Scf" means a high *k*_*s *_rate whereas "wo-Scf" means a zero *k*_*s *_rate. The angular forces were excluded for the simulations plotted in the left panel. When the scaffold proteins are active and thus bind to molecules *A*, a higher concentration of phosphorylated *A *is only measured in the spatial simulation (red line).

By choosing a high *k*_*s *_value now, several particles A bind to the scaffold proteins. Since their diffusion is limited to the surface of the scaffold protein, they can phosphorylate each other with a higher probability than when diffusing in the whole reactor. A higher concentration of phosphorylated A can thus be measured. When switching on angular forces that push scaffold-bound proteins A to one pole, the effect is further amplified. A zero value for *k*_*s *_results in slower phosphorylation of A. When simulating the same model without the inclusion of space in BioNetGen [24], the level of phosphorylation is independent of *k*_*s *_(see Figure 6).

Certainly the same effect could be achieved in non-spatial simulations by adding reactions with higher rates for scaffold bound species. The important difference here is that in the spatial, rule-based approach, a higher phosphorylation rate for bound molecules emerges from the given rules, but is not explicitly defined. It can also be argued, that a phosphorylation occurring between two molecules A that are already part of a complex with the scaffold S, is actually an intra molecular reaction. Hence it would constitute a monomolecular reaction with a completely different reaction rate. However, in our approach, the same bimolecular reaction rate that is measured from freely diffusing particles A can even be applied to the related reaction that happens in a larger complex. Two subgraphs of a complex molecule graph then behave like independently diffusing molecules to the reaction executing algorithm of SRSim (see Figure 1d). Simultaneously, they are linked together for the spatial simulator, which leads to higher interaction frequencies. Ultimately, the effective monomolecular reaction rate is a function of the bimolecular reaction rate and the correct description of the particle geometries and the diffusion on the scaffold molecule's surface. Practically, it might still be at least as complicated to determine the correct geometry parameters as to measure the monomolecular reaction rate experimentally. The other way round, geometric properties might be estimated from bimolecular reaction rates or might even be reusable for different molecular species.

### Growth of Filaments and Active Transportation

**Figure 7:**
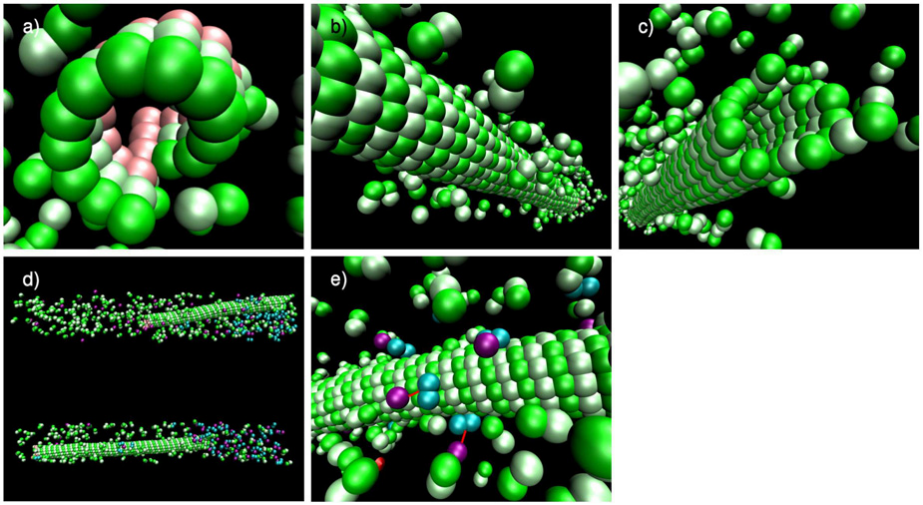
**Active transportation along microtubules**: 600 filament dimers (green/light green) and a nucleating structure (pink) are initially added equally distributed to the reactor (a). These self-assemble into a microtubule (MT) with a 13_3 helix, meaning there are 13 protofilaments and a helicity of three dimers per turn. As a result, a seam forms where *α*-tubulins bind to *β*-tubulins laterally (b). Association and dissociation processes constantly happen at the plus end of the MT (c). 50 motor protein dimers (cyan) and 50 cargo particles (purple) are added in the second phase of the simulation. Initially the cargo is equally distributed in the reaction volume (d, top). Finally, a high concentration of cargo particles is created by active transportation at the plus end of the MT (d, bottom). Motor proteins that have already bound to cargoes can bind to the MT as well and start moving to the plus end (e).

ATP driven transportation of cargo molecules along the tracks constituted by microtubules (MTs) or actin filaments has an important impact on the function of many processes from intracellular material transport to muscle contraction and cell division [15,63-65]. In this context, a simplified model demonstrates the possibility of using the SRSim system to describe the self-assembly and function of complex intracellular structures and molecular machines. A hollow tube representing a microtubule is first grown in the simulation and then used as a track for motor proteins like kinesin- or dynein proteins, which move cargoes from one end of the reactor to the other (Figure 7).

In the first simulation phase, *α*- and *β*-tubulin heterodimers are assembled to form a tube of 13 protofilaments, starting from a cyclic set of capping molecules at the minus end. The biological counterpart to this structure is the *γ*-Tubulin-Containing Ring Complex (*γ*TuRC) in the Microtubule-Organizing Centers (MTOCs) [66]. Eventually, a 3-start helix is formed with a seam as it can be observed *in vivo *and *in vitro *[15,67,68].

In the second phase, motor proteins are added to the simulator, which can bind to the microtubules and to a heavier freight molecule. Like kinesin [69], the motor proteins move along the MTs in the direction of the growing plus-end of the MT. Motion is generated by binding and breaking bonds between the tubulin and kinesin dimers. One part of the motor always stays attached to the MT, while the other part diffuses to bind to the next position of the MT lattice [63,70-72].

The model description comprises 27 rules: 13 of them for the polymerization of the microtubule including the seam, four to allow the processive steps of the kinesin motors and another 10 rules to control the binding and release of cargo and the microtubule lattice by the motors.

Elaborate spatial simulations concerning MT dynamics have already been carried out by others [68,73], whereas our microtubule/kinesin simulation was kept simple and was rather used as a toy model to test the simulation software and show its versatility. Nonetheless, it might easily be extended to include effects like dynamic instability [15] or ATP and GTP turnover. It might also serve to evaluate the effect of divergent protein geometries and different models for the assembly and decay of MT lattices on various levels of detail.

### Spheric Self-Assembly

Self assembling, spherical structures in real living cells are for example viral shells [16,17] or PML-Bodies [74,75]. Though the simulations presented here are not intended to be realistic representations of these biological entities, related processes might still be involved in their formation. Hence, this exemplary application focuses on the emergence of macroscopic spherical, self-assembling structures as a result of diffusing, geometrically constrained molecules.

**Figure 8:**
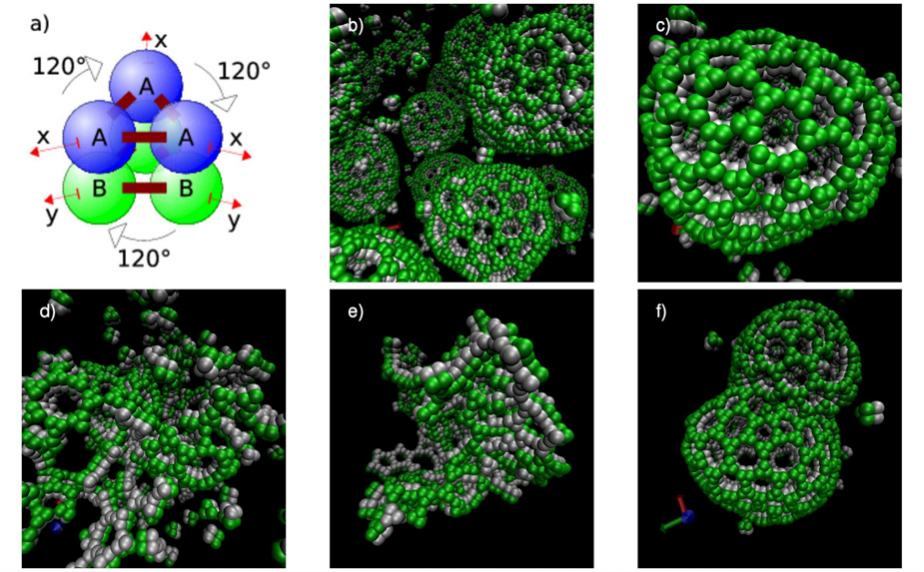
**Self-assembly of spheric structures**: The used monomer geometry is displayed in panel (a). All six components of the complex are handled together as a rigid body, so no force calculations have to be performed among the components themselves. It is composed of a triangle of "outer" components of type *A*, which will later be at the outer side of the spheres and a triangle of "inner" components of type *B *forming the sphere's inside. Some assembled spheres of approximately the same size can be observed (b). Viewing one of them closer, the forming pentagons and hexagons are visible (c). When using dissociation rates a bit too high, small cyclic assemblies decay too fast to form whole spheres (d). On the other hand, when the dissociation rates are chosen too low, static, mal-formed complexes emerge (e). Occassionally, the separation of a big sphere into two smaller ones can be observed (f).

The employed "monomers" are more complex now, being composed of six elementary molecules of two different types (see Figure 8, a). We use the notion of monomers here, because there is no reaction rule in this simulation that can further disassemble the basic complex of six elementary molecules. Consequently, to save computational resources, the compounds of our monomers are treated as rigid bodies by the spatial simulator. When two monomers are connected, this can only happen between two components of the same type. Hence, to obtain the curvature of the spheres, the bond lengths (x) of the "outer" components are set to a distance of 1.4 units, while the bond distances for the inner components (y) are set to 1.0. Varying these distances, different sized spheric structures can be obtained. Due to the modeled flexibility of the molecules, the resulting sphere diameters are not fixed, but lie within a certain range of values.

To describe the self-assembly process, eight rules and four parameters are used in this example. They are organized in pairs, as there is always one rule for the "inner" and one for the "outer" components. While the first pair of rules describes the coupling of two free monomers, the next pair handles the more likely event of the addition of a monomer to an already formed complex. The two remaining rule pairs specify the dissociation behavior.

When the on- and off-rates are chosen carefully, the dynamic formation of cyclic pentamers and hexamers can be observed in an early simulation phase (Figure 8, d). Later on, larger assemblies close into spherical complexes (Figure 8, b, c), which occasionally form and close fissures, leading to the exchange of particles with the environment. While a regular hexagonal lattice would create a planar structure and a regular assembly of pentagons created an dodecahedron, the spheres observed here irregularly accommodated cycles of five, six or seven monomers.

In contrast to the tubular structure of the microtubules in the last example that was predetermined by the capping molecules, the formation of these spheric structures is an emergent effect of the monomer geometry and flexibility. The spatial rule-based approach might be used to help in the analysis and formation of hypotheses concerning the assembly pathways, kinetics and geometries of related problems.

#### DNA Sierpinski Triangles

Another short example from the area of biomolecular computing [76] shows a simplified *in silico *reproduction of the self-assembly of Sierpinski triangles from DNA-tiles [77]. The original experiments were conducted by Rothemund and co-workers [18], highlighting the similarity of the process to the function of a cellular automaton calculating the XOR function ⊕.

**Figure 9:**
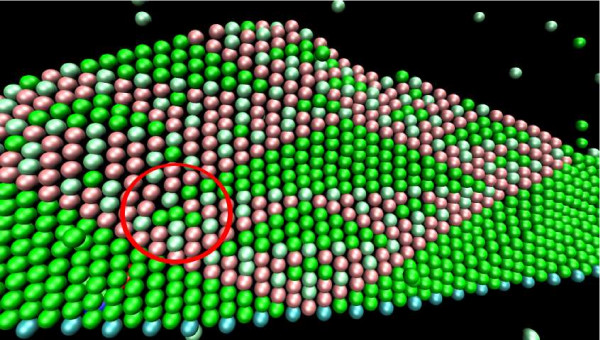
**Results of a tile assembly simulation with SRSim**: Sierpinski triangle structures can be observed as well as an assembly error in the marked circle. The cyan molecules at the bottom line represent the nucleation structure. Molecules in green and light-green represent '0' tiles, while the red ones denote '1' tiles. Green and light-green molecules are different, as the first ones can only dock to two '0' tiles, while the latter ones can dock only to two '1' molecules.

**Figure 10:**
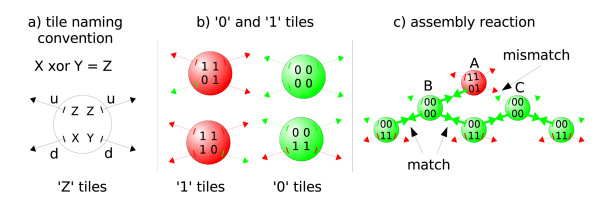
**Monomers representing DNA tiles**: Each DNA tile can have four connections to other tiles. They are made of single stranded sticky ends in Rothemund's experiment using hybridization of real DNA and are represented as binding sites in our experiment with SRSim. Two binding sites *d *are oriented downwards, two other components *u *upwards (a). Green arrows set out upwards from a '0' tile, whereas red arrows leave '1' molecules (b). To connect two tiles, a component *u *from a lower tile can bind to a component *d *from an upper tile if they are in the same color. The attachment of a new tile (c) happens in two steps. When a '1' tile *A *has bound to a '0' tile *B*, it can bind to a '1' tile later. But if the other tile *C *represents a '0' tile, no second connection stabilizes the bond between *A *and *B*, leading to a soon dissociation of tile *A*.

The calculation happens by asynchronous addition of layers of DNA-tiles on "top" of a one-dimensional nucleating structure (see Figure 9). Considering the binary XOR function ⊕, there are four types of DNA-tiles corresponding to the truth table entries (0 ⊕ 0 = 0, 0 ⊕ 1 = 1, 1 ⊕ 0 = 1, 1 ⊕ 1 = 0, see Figure 10).

Basically every DNA tile to be added to a present assembly should be connected to two other tiles. But since we can only form one new connection in a single reaction step (see Section Methods), the effective trimolecular reaction is separated into two distinct bimolecular reactions (see Figure 10, b).

Although there are simulation techniques more specialized in tile-based DNA self-assembly [78], we think that spatial rule-based simulation might help planning experiments also from the field of biomolecular computing by examining various sources for errors *in silico *on different levels of detail.

While the possible interactions between the DNA-tiles are described on the level of rules in the presented simulation, it might for example be interesting to use individual nucleotides. Only two rules would then be necessary to implement a Watson-Crick complementarity at the expense of more detailed DNA-tiles.

## Discussion

### Higher Order Reaction Kinetics

Because we simulate individual, diffusing particles, the number of collisions between the particles approximately leads to mass action kinetics. But how could more elaborated kinetic laws like Hill or Michaelis-Menten kinetics be realized? Though these would not typically be used in particle simulations, many biomodels are specified relying on these laws. Higher order kinetics also constitute a valuable tool to simplify reaction systems, e.g. by saving the trouble of explicitly simulating otherwise irrelevant enzymes. The main problem for the use of these kinetics is probably the measurement of concentrations that are included in the kinetic law's formula. While our approach uses information about individual particle's (stochastic) behavior, a concentration dependent law would need the particle density counted in a subvolume. The size of this volume lies in between the two extremes of the whole reactor and tiny volume elements hardly containing single particles. Then, the elaborated kinetic laws could dynamically change the propensities of the dependent reactions.

### Analyzing Simulation Runs

How can simulation runs of spatial and rule-based systems be analyzed? Next to counting the numbers of molecules in the reactor or regions of it, also the appearance of certain reactant patterns seems important. Different approaches as well as our own use this technique up to a certain extent [21,79]. For example, one might be interested in the amount of tubulin dimers, that have bound GTP and both lateral neighboring tubulin proteins. However, many different modes of counting patterns are conceivable, as for instance imagine a complex molecule being composed of three elementary molecules *m*_*A *_- *m*_*B *_- *m*_*A *_from two elementary species. Dependent on the mode of counting, the observed pattern *m*_*A *_- *m*_*B *_can now be found either once or twice in the complex molecule graph. Hence combinatorial effects have to be considered carefully.

### Simulation Timescales

At the moment, when using single desktop PCs, we can achieve simulated times in the order of some milliseconds. When assuming a system of about 10 thousand particles, a simulation for 3 * 10^6 ^time steps took one of our desktop PCs about four hours.

When we consider a protein in the size of the hemoglobin tetramer with a diameter of about 5.5 nm, its diffusion coefficient should be in the range of . Here we used the Stokes-Einstein relation [27], with the Boltzmann constant *k*_*B*_, the absolute temperature *T*, the solvent's viscosity *η *and the particle's radius *r*. The error in the kinetics should be quite low when we choose our time step Δ*t *so that the mean expected translation of a particle  is about a 10th of the particle diameter. That would result in a time step of about 0*:*5*ns *and a total simulated time of 1.5 milliseconds.

Though the scale of some thousand particles and some milliseconds may be sufficient only for special problems, the SRSim approach will be applicable in more complex situations as well. In our simulations, the bottleneck of the computations is the calculation of forces and the displacement of the particles, but not performing the reactions. Given we do not want to simplify the spatial model, computers are still becoming faster and use dedicated hardware like parallel graphic processors (GPUs). Molecular dynamics systems are predestined for parallel computations since the processes in the reactor can be split up into independent, local sub-problems. The simulator LAMMPS that underlies SRSim is already fully parallelized via the Message Passing Interface (MPI) and can be run on large computer clusters.

Another means to speed up the computations is to abstract away aspects of the simulation that do not seem important for a certain system. Inflexible molecule graphs and delocalized particles can be used in an event-based simulation as done in the self assembly simulator by Zhang et al. [46]. The focus is on the formation and structure of complex macromolecules instead of the positioning or the elasticity of the molecules. A different approach would be to preserve information about local dynamics within a complex molecule, but speeding up the calculations by running embedded simulations. That would lead to an efficient stochastic model for the conformations of large but stable complexes. Larger time steps might also be used by sacrificing the detailed brownian movement of every particle. Green's Function Reaction Dynamics [80] calculates the time until the next reaction occurs from the positions of all molecules at a time step. Then all the particles can be displaced by this dynamic time step instead of having many smaller time steps that involve no reactions. Nonetheless, this technique would ignore the effects of steric hindrance between complex molecules. Systems involving small molecules like ATP occurring in high amounts could be simulated as continuous concentrations in the background to relieve the particle-based simulation engine of high particle numbers. Similar separations of scales were used in the simulator Cell++ [37].

### Implications of the Chosen Geometric Level of Detail

In our current exemplary implementation of a spatial, rule-based reaction system, we chose a level of detail that allows angles but no dihedral angles. Here, with angles we denote the angle between a central molecule *m*_*i *_and two further molecules *m*_*j*_, *m*_*k *_adjacent to *m*_*i*_. Dihedral angles describe the torsion around the axis *m*_*j *_- *m*_*k *_, if the molecules *m*_*i*_, *m*_*j*_, *m*_*k*_, *m*_*l *_are linearly connected. Alternatively, dihedral angles can be imagined as the angle between a plane *p*_1 _through the first three molecules *m*_*i*_, *m*_*j*_, *m*_*k *_and another plane *p*_2 _through the second three molecules *m*_*j*_, *m*_*k*_, *m*_*l*_. Consequently, it is not trivially possible to specify or inhibit the torsion around a bond. This would for example be necessary to distinguish a helix as displayed in Figure 3(e) from a circle in a single plane. To specify complicated geometries in the present level of detail, one could employ combinations of elementary molecules as building blocks, as it was done in our spheric self-assembly simulation.

Another aspect of our chosen level of detail is that unbound elementary molecules (EM) do not posses a rotational orientation. Hence, the molecule is expected to rotate diffusively so that all possible orientations are considered to be equally possible for the calculation of geometric compatibilities. This treatment of single EM is similar to orientation-less particle models as used in approaches by Gillespie [59] or ChemCell [44]. As soon as an EM *m*_*i *_is bound in a complex molecule graph, there are bonds that realize component vectors of *m*_*i*_. When a new bond to an EM *m*_*j *_should be formed, this is only allowed if the angles between the present component vectors of *m*_*i *_and a potentially new component vector pointing towards *m*_*j *_are deviating less than the tolerance value *t*_*ang *_from the ideal bond angles. In other words, this means that the reactive volume  to bind another EM *m*_*j *_can change, dependent on other EM that are connected to *m*_*i*_. This has to be considered when specifying reaction rates.

## Conclusions

The spatial, rule-based simulation approach, represented by our exemplary tool SRSim, constitutes a versatile, rule-based simulation system, which accounts for inhomogeneous distributions, volume-exclusion, structure and geometry of diffusing particles. It is able to tackle a variety of problems from the scope of self-assembling biomolecules and molecular processes exhibiting combinatorial complexity. For some of these areas problem-specific simulation systems have already been developed [16,17,68,73,78,81]. Our approach might help to integrate the description and treatment of many such problems under a common, effective formalism.

Though molecular dynamics studies of interacting proteins are considered the most physically accurate representation of biochemical processes [10,82], they are not always achievable or desirable. The computational effort is immense even for only two involved macromolecules, and high resolution 3D structures have to be known for each involved particle. In contrast, the level of geometric detail in spatial rule-based models can be chosen dependent on the present level of knowledge and the aspired scale of the system under consideration. Similar to Coarse Grained Molecular Dynamics [83,84], one spheric particle of the simulation can represent anything from a single atom to a big macromolecular complex.

Another important point of our approach is the fact that the dynamics of reaction networks can quantitatively and qualitatively change as an effect of heterogeneous spatial concentrations as well as in dependence of the employed particle geometries. Combinations of structural parameters for the involved particles as component angles, distances, component tolerances and reaction rates can be varied, trying to reproduce observed macroscopic behavior. Thus, possible candidates for macromolecule geometries and hypotheses on the cooperation of complex system's compounds are generated for an efficient experimental evaluation. Let us assume a simple polymerization of identical monomers with two binding sites. In each step, any two monomers could be connected. But whether they form complexes as cycles, rod shapes, helices or something unstructured depends on the geometries of the particle's binding sites (see Figure 3). When considering all reactions that were possible based on the rule-based system, the geometric properties can implicitly disallow or inhibit a subset of reactions and favor others. Some binding sites may be blocked by particles, while others may be co-localized or brought close together, practically forcing a reaction. Though this is not the focus of this paper, also the shape of the reaction container can severely influence the reaction kinetics [45].

Eventually, the dynamics may then diverge drastically from reaction-diffusion systems that consider space by using Partial Differential Equations or spatial variants of Gillespie's algorithm [40,41,52]. In particular, a set of chemical species that can in principle form a stationary state in a reaction-diffusion model derived from a rule-based reaction system may not have this property when geometry is considered. This implies that applying algebraic methods like elementary mode analysis [85] or chemical organization theory [86] to the reaction rules while neglecting geometric information can lead to misleading results. The organization theory operates on the binary presence or absence of species in a reaction system. An organization is formed by a subset of all possible species, if this subset cannot generate new species but can replenish its own species from itself by means of the system's reactions. For example, a set of species reversibly forming tetrameres as displayed in Figure 3, d) that is not an organization with respect to the reaction rules, is an organization when geometry is also considered, because the geometric effects hinder the formation of further species but allow the formation of the tetramers and their preliminary compounds. Note that, obviously, also a set of species that is an organization given just the reaction rules can become no organization when considering geometry.

In addition to pure geometry, the flexibility of the proteins [68,87] to strain, torsion and bending will influence the rates, pathways and structures of formed complexes. For example in the experiment of spheric self-assembly, the exact structure is not pre-determined but emerges dynamically as a function of geometries and flexibilities.

While we have been highlighting the assets of only the spatial features of SRSim so far, it is the combination with the rule-based reaction system that leads to the modeling strength of our approach. In a conventional but spatial reaction system, it would not be possible to easily model complex and highly structured self-assembly reactions including an almost infinite number of species like in the example of growing sierpinski triangles. Also it would not be possible to describe elaborated processes as the successive binding and dissociation reactions that are necessary for the dynamic description of molecular machines like in the example of our molecular walkers along the microtubules. We want to point out in this paper, that it is necessary to combine both, the flexibility of the rule-based reaction system as well as the spatial simulation technique to describe and model a variety of complex biochemical systems.

Further work will also address the incorporation of more powerful rule types. Patterns that allow wildcards for subsets of molecule types might constitute valuable helpers for the model description as well as rules allowing the exchange of whole subgraphs in molecule complexes.

Next to enhancements concerning the rule evaluation and execution system, also different levels of spatial and geometric detail should be considered. Different ways to a more abstract but faster system were shown in the Discussion Section. When, on the other hand, the level of detail should be elevated, there is a range of conceivable changes to the SRSim system. Torsional angles and binding energies might be included in the geometric model, for instance, or the possibility to break bonds not only because of reaction rates but also as a result of applied forces.

The authors believe that advanced modeling of biomolecular systems in future will necessitate both, the treatment of spatial aspects as well as the ubiquitous combinatorial complexities arising from multiprotein complexes and multistate molecules. Therefore, in addition to more corse-grained or analytic approaches [79], we advocate the use of versatile, spatial rule-based simulation systems like SRSim, specifically designed for these modelling demands.

## Authors' contributions

Conceived and designed the experiments: GG BI TH PD Wrote the software: GG Performed the experiments: GG Analyzed the data: GG BI TL ML TH PD Wrote the paper: GG BI TL ML TH PD All authors read and approved the final manuscript.

## Supplementary Material

Additional file 1 **C++ source files to compile SRSim, including the slightly modified LAMMPS sources**. Click here for file

Additional file 2 **Additional text discussing how to convert macroscopic kinetic rates from non-spatial biomodels to the microscopic reaction rates that are applicable only, when two reacting particles are already within the geometric tolerance values**. Also, possible sources for inaccuracies as the use of the refractory time will be discussed. Click here for file

Additional file 3 **Contains the source files for the simulations presented in the Results Section. Makefiles to run the simulations are readily included**. Click here for file
