# Supplementary material for: Rule-based spatial modeling with diffusing, geometrically constrained molecules
Source: BMC Bioinformatics. 2010 Jun 7;11:307. doi: 10.1186/1471-2105-11-307 (PMC2911456; doi:10.1186/1471-2105-11-307)
Supplement: Additional file 1 — C++ source files to compile SRSim, including the slightly modified LAMMPS sources. [file 1471-2105-11-307-S1.ZIP › docs/Manual.pdf]

Chapter 1

SRSim Documentation

1.1 Installation Instructions

The following installation instruction are addressed to x86 linux users, which are assumed to have Gnu Make and a C++ compiler installed. No tests were carried out using different hard- or software platforms. From now on, all mentioned paths relate to the enclosed CD.

Required Software

Unfortunately the installation of SRSim is not yet fully automatized, so there are some uncomplicated steps to do: In the first place, make sure that the following libraries are present on your system, namely **Xerces-C++**[1], which is required for XML parsing. The other dependancy is the "Message Passing Interface"[2] (**MPI**), a parallel computing standard used by LAMMPS. There are different MPI implementations available.

It is recommended but not necessary to install the software "Visual Molecular Dynamics"[3] (**VMD**) [1], which can be very helpful to visualize molecular trajectories calculated by SRSim.

[1] download from `http://xerces.apache.org/xerces-c/` or use the system's packet manager. Versions 2.7 and 2.8 seem to work fine.

[2] download for example MPICH from `http://www.mcs.anl.gov/research/projects/mpich2/` or use your system's packet manager.

[3] download from `http://www.ks.uiuc.edu/Research/vmd/`

Compiling the Rule System

The next step is to compile the Rule System, to form a library which is later linked together with the LAMMPS sources. As you will have to adjust some values in the makefiles, it is probably a good idea to copy the sources to a local directory that you have got write access to. But some system specific paths have to be set, before the actual compilation can begin. Enter the directory `sources/SRSim/RuleSys` of the attached CD and open the file `Makefile` in your favorite text editor. You will find three variables, `LOCALBIN`, `LIBDIR` and `INCDIR` which should be adjusted fitting to your system.

Then, still in the directory `sources/SRSim/RuleSys`, type `make prepare` to install the necessary headers for the rule system, followed by `make install` to build and install the library `libRuleSys.a` in your library path. Try executing `testSRSim notExistingFile.bngl` from your bash prompt - you are ready to continue with the next step if you see a message looking like:

```
###################
ERROR: cannot open file in BNGRuleBuilder::readBNGFile: ...
###################
```

Compiling the LAMMPS Sources

If the rule system was built successfully, the original LAMMPS sources can now be compiled, together with the additional Modules introduced for SRSim. To facilitate the process, the sources of LAMMPS are already prepared together with the additional modules in the directory:

`sources/SRSim/LammpsCompilation`

Once again, some adjustments will have to be made to the variables defined in the `Makefile`. Probably the most important entry is `DST` for specifying the path where the LAMMPS executable should be placed. If you are doing a local installation or some of your libraries and include files are placed in uncommon directories, the entries `CCFLAGS, LINKFLAGS` and `USRLIB` should be checked. Finally, type `make install` to compile and link the executable. If the command `lammps` outputs the following line, you are done installing SRSim.

```
LAMMPS (5 Oct 2007)
```

1.2 Using the Software

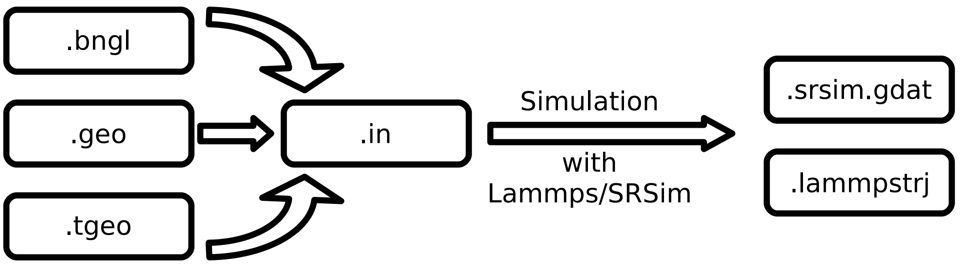

Figure 1.1: Overview on the input / output file structure of SRSim.

Since SRSim is actually no autonomous program, but rather a component for the molecular dynamics simulator LAMMPS, it is started exactly like LAMMPS is: `lammps < input_script.in`
The input script `.in` is then referencing three other input files and two or more output files as illustrated in Figure 1.1. The referenced input files are the `.bngl` file for the used rule-based reaction system, the `.geo` file for the molecular geometry definition and finally, the `.tgeo` file for the template geometry definition. The output files will usually be a `.srsim.gdat` file containing the concentrations of the observed species and a `.lammpstrj` file with all the molecular coordinates, which can be used to visualize the simulation run. Unfortunately there is no graphical user interface yet, so the input files have to be managed manually.

To analyze the results, gnuplot and vmd can be used. Type `vmd output.vmd` for instance, to see a graphic representation of the reaction volume. Since VMD (See Section 1.1) was rather designed to display all-atom systems, the results are not ideal. In my opinion, the best images are obtained using the "Van-der-Waals"[4]

The LAMMPS Input Script

The LAMMPS input script is parsed line by line, each of which holds one command modifying the simulation system. Comments can be added using the # sign. Note that the order of the commands is important since the input script is parsed line by line. There is a large number of possible commands

[4]In the main VMD window select the menu "Graphics->Representations...", than select "VDW" from the drop-down menu "Drawing Method".

that can be used to customize the simulation, so please refer to the LAMMPS documentation[5] for further details on the original LAMMPS commands.

In the first phase, some basic parameters have to be set, as for example the units to be used, the desired atom style, the size of the reaction volume, the maximum number of molecular species and the initial configuration of the simulation system. Note that the command `atom_style` uses a special atom style, specially designed for SRSim, which is followed by the names of the further input files and the random seed.

Listing 1.1: input_script.in - part 1

```
dimension      3
boundary       f f f       # use fixed boundary conditions
units          metal       # timescale: ps,  distances: Angstrom
newton         on
atom_style     srsim  input.bngl  input.geo  input.tgeo      11111
##########     cmd      .bngl        .geo       .tgeo    random_seed

lattice        none
region         Nucleus block 0 1000 0 1000 0 1000 units box
######                   dimensions of the reaction volume

create_box         100           Nucleus
##########         n_atom_types  Region_name

neighbor           50.0 bin
start_state_srsim  coeffs     5.0e-3        5.0e-3       5.0e-3
#################  cmd        fRepulsion    fBond        fAngle

start_state_srsim  atoms            #   add the molecules to the sim
```

In the next phase, different "fixes" are selected to be applied to the simulation. These are computations which influence each molecule's data, for example their positions, velocities or binding states. The most basic fix, called "`nve`", is the calculation applied to move each particle according to Newton's equations of motion in dependancy on the applied forces. The fix `langevin` adds implicit solvent effects, resulting in brownian movement of the particles. The second last parameter to the fix `langevin` is called damping factor (γ^{-1}). It depends directly on the diffusion coefficient. Since fixed boundary conditions were chosen before, molecules moving out of the reaction volume would be lost, so the fix `wall/reflect` is applied. The last fix, `srsim` is the part of SRSim that checks for molecular collisions, analyses which rules are applicable and finally executes these.

Listing 1.2: input_script.in - part 2

[5]The LAMMPS documentation comes together with LAMMPS' sources and can be accessed online at `http://lammps.sandia.gov/doc/Manual.html`.

```
fix  1   all   langevin  300  300  2700.0   23456
#        parameters:     Temp Temp Gamma^-1 random_seed

fix  2   all   nve
fix  3   all   wall/reflect xlo xhi ylo yhi zlo zhi

fix  4   all   srsim   1   45678  4.7    5e-2 1.0 5e-2 1.0 40
#  fix srsim syntax: fix id group srsim | nEvery  randomSeed  preFactBindR
#                                         preFactBreakR preFactExchangeR
#                                         preFactModifyR_1 preFactModifyR_2
#                                         refractoryTime
```

In the last phase, the types of output and the length of the simulation runs will be defined. The dump type `srsim` creates a plain text file in the same format as BioNetGen, to allow an easy comparison of the computed trajectories. Note that the intervals between two successive output data writes can be changed using the command `dump_modify`. If new molecules to be added to the running simulation, the command `runmodif_srsim addMols` can be used, given the specified molecule-graph type was already listed in the reaction system definition.

Listing 1.3: input_script.in - part 3

```
timestep     10.0
thermo       500    # output thermodynamics output all 500 ts

dump         1  all   srsim 500    output.srsim.gdat
dump         2  all   atom  5      spass1.lammpstrj
#            id group type  nEvery fileName

dump_modify 2 scale yes

# record an exact trajectory for the first 1000 ts:
dump_modify     2 every 1
run             1000

# record only 500k-th frame for the next 20mio ts :
dump_modify     2 every 500000
run             20000000

# add 2000 molecules of P31 between the coords (400,800,2500) and
# (500,900,2600). Then simulate for another 5mio ts:
runmodif_srsim  addMols 400 500 800 900 2500 2600 P31(m2) 2000
run             5000000
```

The Reaction System File

The rule-based reaction system is specified in the BioNetGen Language (BNGL, See the BioNetGen Documentation[6]). Basically it is not necessary to change a BNGL file to use it with SRSim. However, the commands `generate_network` and `simulate_ode / simulate_ode` at its end will be ignored. In addition, the more important command `setConcentration` will be ignored, which is used to change some species' concentration during the run. This command's behavior can be approximated by using `runmodif_srsim` in the LAMMPS input script instead.

The Molecule and Template Geometry File

In order to communicate the properties of all the different molecular species to SRSim, the geometry files `*.geo` are used. For each species, the mass and radius as well as the attributes for each site have to be defined for an introduction to the geometry model). Furthermore, the site tolerances are also specified in this file, which are not yet definable for each site individually. Since the syntax of the `*.geo` files is the common XML format, only an example is presented.

Listing 1.4: input.geo

```
<?xml version="1.0"?>
<molecule-geometry-definition>

  <ReaktionGeometry>
     <DistanceDeviation value="50" />
     <AngularDeviation value="120" />
  </ReaktionGeometry>

  <molecule name="Mad2" >
     <mass   value="30000.0" />
     <radius value="30.0" />
     <site name="m1"    phi="0"   theta="0"     dist="30.0" />
     <site name="m2"    phi="0"   theta="109.5" dist="30.0" />
     <site name="cdc20" phi="120" theta="109.5" dist="30.0" />
     <site name="conf"  phi="240" theta="109.5" dist="30.0" />
  </molecule>

  <molecule name="Cdc20" >
     <mass   value="35000.0" />
     <radius value="35.0" />
     <site name="m2" phi="0" theta="0" dist="35.0" />
  </molecule>
```

[6] A BioNetGen tutorial can be found online at `http://bionetgen.org/index.php/BioNetGen_Tutorial` .

```
</molecule-geometry-definition>
```

When whole molecule graphs are added to the simulation, the relative positions of all the molecules have to be known. So they are calculated in advance by an independent simulation step using `createGeo` and stored in the `*.tgeo` files. Here again, the XML format is used, so manual changes in the template configuration is still kept possible.

Listing 1.5: input.tgeo

```
<?xml version="1.0"?>
 <template-geometry-definition>

    <template id="0" name="Mad1(m2!1).Mad2(m1!1,m2,conf~Closed,cdc20)">
       <mol id="0" x= "26.5957" y="-5.54135" z= "4.23955" />
       <mol id="1" x="-26.5957" y= "5.54135" z="-4.23955" />
    </template>

 </template-geometry-definition>
```

The Tool "createGeo"

To simplify the creation of `.geo` and `.tgeo` files, the tool `createGeo` was added to the SRSim programs. It is used in the following syntax:

`createGeo input.bngl input.geo input.tgeo`

If either the `.geo` or the `.tgeo` file is not exiting, it will created. Molecule geometries are created with initial values of 1.0 for all distances and predefined angles for up to 6 sites. Template geometries calculated by running short MD simulations to relax all bond distances and angles.

Bibliography

[1] William Humphrey, Andrew Dalke, and Klaus Schulten. VMD – Visual Molecular Dynamics. *Journal of Molecular Graphics*, 14:33–38, 1996.
