# Supplementary material for: Rule-based spatial modeling with diffusing, geometrically constrained molecules
Source: BMC Bioinformatics. 2010 Jun 7;11:307. doi: 10.1186/1471-2105-11-307 (PMC2911456; doi:10.1186/1471-2105-11-307)
Supplement: Additional file 2 — Additional text discussing how to convert macroscopic kinetic rates from non-spatial biomodels to the microscopic reaction rates that are applicable only, when two reacting particles are already within the geometric tolerance values. Also, possible sources for inaccuracies as the use of the refractory time will be discussed. [file 1471-2105-11-307-S2.PDF]

Supplement: Rule-based spatial modeling with diffusing, geometrically constrained molecules

Gerd Gruenert Bashar Ibrahim Thorsten Lenser Maiko Lohel
Thomas Hinze Peter Dittrich

January 14, 2010

Calculation of Kinetic Parameters from Macroscopic Values

When assuming a well stirred reactor and known macroscopic kinetic rates for mass action kinetics, microscopic rate constants for SRSim can be calculated. Prerequisite is knowing the characteristic geometric tolerances for the formation of bonds. In many cases, there will be no values available from biophysical or theoretical experiments yet. Even having generated a certain macroscopic behavior with fitted parameters in a simulation experiment does not prove that the physically correct parameters are found. There might still be various microscopic parameter combinations that would generate this effect. Nonetheless, no hypothesis can be verified until it is formulated. So different parameters can still be tested for their influence on the model and various scenarios about possible microscopic explanations for a macroscopic effect can be evaluated.

Beginning with monomolecular reactions like $A \xrightarrow{v_1,k_1} B$, a macroscopic rate constant k_{1mac}, given in s^{-1}, is translated to k_{1mic} by expressing the reaction rate in terms of the expected number of particles $E(N_{\text{reacting in dt}})$ undergoing the reaction in an infinitesimal time step dt. Then, the reaction rate v_1 equals

$$v_1 = c_A\ k_{1mac} = \frac{N_A}{V_{reactor}\ \eta} k_{1mac} \overset{!}{=} \frac{E(N_{\text{reacting in dt}})}{V_{reactor}\ \eta\ dt}$$

with the concentration c_A and the number of particles N_A of species A in the reactor of the volume $V_{reactor}$. Please note that we named the Avogadro constant η instead of N_A to avoid misinterpretation with the particle numbers. The expected number of reacting particles is the sum over the N_A infinitesimal probabilities $k_{1mic}dt$. Each is giving the probability for a particle of type A to react in the infinitesimal small time step dt:

$$E(N_{\text{reacting in dt}}) = \sum_{i=1}^{N_A} k_{1mic}dt = N_A k_{1mic}dt$$

Hence $k_{1mic} = k_{1mac}$.

The reaction propensities for bimolecular reactions can be calculated in almost the same manner. Assuming a well stirred reactor again, the speed of a reaction $A + B \xrightarrow{v_2, k_2} C$ is expressed as the expected number of bonds formed between the elementary molecules A and B. This number has to correspond to the macroscopic reaction rate.

$$v_2 = c_A \ c_B \ k_{2mac} = \frac{N_A \ N_B}{V_{reactor}^2 \ \eta^2} k_{2mac} \stackrel{!}{=} \frac{E(N_{\text{reacting in dt}})}{V_{reactor} \ \eta \ dt}$$

The expectation value for the number of reacting particles $E(N_{\text{reacting in dt}})$ is the sum over all possible pairs of molecules A_i and B_j of the product of the microscopic reaction propensity, the infinitesimal time step dt and the probability for two molecules to be positioned compatibly to each other. Please note that

$$\begin{aligned} E(N_{\text{reacting in dt}}) &= \sum_{i,j}^{N_A, N_B} k_{2mic} dt \ P(A_i \text{ and } B_j \text{ geometrically compatible}) \\ &= N_A \ N_B \ k_{2mic} dt \ P(A_i \text{ and } B_j \text{ geometrically compatible}) \end{aligned}$$

The probability for any molecule A_i to be positioned in geometric compatibility to a molecule B_j in our simulator is governed by the particle's geometric tolerance values. These define the reactive volumes V_A, V_B for the elementary molecules (See Section Methods and Figure 4). Now A_i has to lie in V_{B_j} while B_j also has to lie in V_{A_i}. Assuming a macroscopically well-stirred reactor, the probability for this to happen is directly related to the fraction of the reactor's volume that the reactive volumes V_{A_i} and V_{B_j} take up.

$$\begin{aligned} & P(A_i \text{ and } B_j \text{ geometrically compatible}) \\ & \quad = P(A_i \ in \ V_{Bj}) * P(B_j \ in \ V_{A_i} | A_i \ in \ V_{B_j}) \\ & \quad = P(A_i \ in \ V_{Bj}) * P(B_j \text{ oriented towards } A_i) \\ & \quad = \frac{V_{B_j}}{V_{reactor}} * P(B_j \text{ oriented towards } A_i) \\ & \quad = \frac{\frac{2}{3}\pi(d_0 + t_{dist})^3(1 - cos(t_{ang})) - \frac{2}{3}\pi(d_0 - t_{dist})^3(1 - cos(t_{ang}))}{V_{reactor}} * \\ & \quad P(B_j \text{ oriented towards } A_i) \\ & \quad = \frac{4}{3} \frac{\pi(3 \ d_0^2 \ t_{dist} + t_{dist}^3)(1 - cos(t_{ang}))}{V_{reactor}} * P(B_j \text{ oriented towards } A_i) \\ & \quad = \frac{4}{3} \frac{\pi(3 \ d_0^2 \ t_{dist} + t_{dist}^3)(1 - cos(t_{ang}))}{V_{reactor}} * \frac{1}{2}(1 - cos(t_{ang})) \\ & \quad = \frac{2}{3} \frac{\pi(3 \ d_0^2 \ t_{dist} + t_{dist}^3)(1 - cos(t_{ang}))^2}{V_{reactor}} \end{aligned}$$

We assume that the orientation of B_j is uniquely specified through its other bound components. If this is not the case, different formulations for the volume of the reactive volume

have to be used. This implication of the level of detail that was chosen for our exemplary implementation of the spatial and rule-based approach is further described in the Discussion Section. Now, V_{Bj} has the volume of a spherical cone with the radius $d_0 + t_{dist}$ minus the lower bound spherical cone of the radius $d_0 - t_{dist}$, where d_0 is the ideal bond distance and t_{dist} is the distance tolerance for bonds. It has to be considered, that once B_j is in V_{A_i} then A_i is already at the right distance to B_j, but can still be oriented into the wrong direction. So the probability for A_i to be facing B_j is the surface of a unit sphere's dome with an opening angle of the angular tolerance setting t_{ang} divided by the surface of the whole sphere. Eventually, k_{2mic} can be calculated as:

$$k_{2mic} = \frac{k_{2mac}}{V_{reactor}\ \eta\ P(A_i \text{ and } B_j \text{ geometrically compatible})}$$
$$= \frac{3\ k_{2mac}}{2\pi\eta(3\ d_0^2\ t_{dist} + t_{dist}^3)(1 - cos(t_{ang}))^2}$$

As we mentioned before, the calculated rates are valid for well-stirred reactors only. This does not have to be true for a system that is described with diffusing particles. As a result, the effective macroscopic reaction rates of the simulated system might be lower than expected, if the particles are not diffusing fast enough.

Applicability of the SRSim Approach

The Gillespie Algorithm [1,2] correctly simulates the time evolution of a conventional chemical system - without complex geometric constraints - in a well-stirred reactor. Here "correctly" means that the algorithm produces a realization of the probability distribution given by the chemical master equation of the system. One of the most important questions for SRSim is how accurate this approach can sample system states from the ensemble of a combinatorial complex biochemical system, when time, space and geometries are considered. We certainly have the problem of introducing many new parameters for the geometry setup of our reacting particles. These may be unknown for a large number of biochemical compounds. Even Molecular Dynamics simulations including all atoms, which are considered the most accurate spatial simulation technique [3] have the problems of containing too many parameters and of using force field approximations. By going to a more coarse description of a system, we can save computational time and abstract away unknown parameters, e.g. the exact 3d-structure of a protein. But we are losing details that we might be interested in on the other hand. If we do not know about geometry parameters for a system's particles - or we do not want to experiment with them, we cannot obtain information about the structure of forming macromolecular complexes.

A possible source for inaccuracies in SRSim is choosing the time step too high. From the kinetics point of view, the positions of the particles in the reactor are fixed during the whole time step $[t, t + \triangle t[$ and change instantly when the time is incremented for the next time step. A reaction can occur if the involved reactants are located within each others *reactive volumes* (See Figure 4). When the time steps $\triangle t$ are chosen too high, a particle might miss

its chance to react because it instantly moves past the reactive volume of another particle. On the other hand, if it was located in the reactive volume, it does not have the possibility to leave the reactive volume. A "real" spherical brownian particle on the other hand would search through a volume of $8\pi DR\triangle t$ before being translated by a distance of $\sqrt{6D\triangle t}$ [4], with the diffusion coefficient D and the particle's radius R. From the physics point of view, also the forces that are acting on a particle between two timesteps are only approximated. If timesteps are chosen too long, for example bond vibrations can escalate.
As a rule of thumb, $\triangle t$ should be chosen so, that the expected translation of a particle $\sqrt{6D\triangle t}$ should be within a 10th of the particle's diameter. To verify whether the chosen time step is sufficiently small, a practical method is to run two other simulations. One with half the step site $\frac{\triangle t}{2}$ and another one with the double step size $2\triangle t$. If the results diverge "too much", smaller time steps should be used [5, 6]. To completely eliminate the error to the reaction rate made by accepting "frozen" particle positions during the $\triangle t$ intervals and by using reactive volumes, the probability function for particle positions and the expected number of collisions between diffusing molecules might be calculated more accurately by using approaches like Green's Function Reaction Dynamics (GFRD) [7, 8]. Implications of rotational diffusion and non-uniformly reactive particles are discussed in [9–11].

A second possible reason for errors is the use of the *refractory time*. We introduced the refractory time to reduce the computational effort of a large number of ineffective, successive binding and dissociation reactions (see Section Methods). For well-stirred reaction system, this effect is usually ignored. When a decay reaction separates two subunits of a complex, both parts are considered to be separated fast enough, so that they can interact with any other molecule with the same probability. By including space in the simulations, a broken bond between two elementary molecules can be formed again before both particles are separated by diffusion. This process is called a *geminate recombination* [12]. Its probability depends on the size of the used reactive volumes, which is described by the geometric tolerance values. To use larger time steps, as seen before, larger reactive volumes might be chosen. But this also increases the number of ineffective dissociations. To reduce those geminate recombinations, the refractory time t_{ref} should be chosen long enough, so that the expected diffusive movement ($\sqrt{6Dt_{ref}}$) of both particles can move them out of each others reactive volumes. Analogous to the notation of SmolDyn [6], we denote the probability of geminate recombination with ϕ. So ϕ is the probability for a binding reaction to reconnect a pair of molecules that was split up just before. Let us consider a simple reaction system:

$$A + B \underset{k_{-1}, v_{-1}}{\overset{k_1, v_1}{\rightleftarrows}} C, \qquad K = \frac{[C]}{[A][B]} \stackrel{?}{=} \frac{k_1}{k_{-1}}$$

where k_1, k_{-1} are the kinetic constants for the forward and for the backward reaction respectively, v_i are the reaction rates and K is the equilibrium constant. A and B are the subcomponents of a complex species C. To estimate ϕ in a simulation experiment, a first simulation S_1 can be run with the original parameters leading to the equilibrium constant K_1. While this K_1 should ideally be $\frac{k_1}{k_{-1}}$ in a well-stirred reactor, K_1 might still be influenced by the fraction of geminate recombinations ϕ here. A second simulation S_2 of the

system can then be run with a much higher refractory time. While S_1 contains geminate recombinations, the reactants have enough time to diffuse away in S_2. If the system is not well-stirred, S_2 should produce the theoretically obtained equilibrium constant $K_2 = \frac{k_1}{k_{-1}}$. ϕ can now be calculated from the decreased equilibrium constant K_2.

$$K_2 = \frac{(1-\phi)[C_1]}{[A_1][B_1]} = (1-\phi)K_1 = \frac{k_1}{k_{-1}} \quad ; \quad \phi = 1 - \frac{K_2}{K_1} = 1 - \frac{k_1}{k_{-1}K_1}$$

Except from geminate recombinations, the use of a refractory time does not further influence the equilibrium constant K, but slightly reduces the total number $[A]+[B]+[C]$ of non-refractory molecules in the system. We assume the following initial concentrations $[C] = [C^0]$, $[A] = [B] = 0$, leading to the conservation constraint:

$$[C^0] = [A] + [C] = [B] + [C]$$

The reaction system given above is now transformed into the slightly more complex system using the transiently refractory species $A^\star$ and $B^\star$:

$$A + B \xrightarrow{k_1, v_1} C, \quad A + B \xleftarrow[v'_{-1}]{} A^\star + B^\star \xleftarrow[k_{-1}, v_{-1}]{} C, \qquad [C^0] = [A] + [A^\star] + [C] = [B] + [B^\star] + [C]$$

$A^\star$ and $B^\star$ are not reacting probabilistically to A and B, but each of these particles changes its state after exactly the refractory time t_{ref}. Eventually, the concentration of the refractory molecules equals $[A^\star] = [B^\star] = t_{ref}v_{-1} = t_{ref}k_{-1}[C]$. To calculate the number of refractory molecules in the equilibrium state, imagine that we can mark all newly produced refractory molecule $A^\star, B^\star$ starting from a time t_i. We are producing these molecules with a rate of $k_{-1}[C]$. When the first molecule that were marked will decay to A and B at the time $t_i + t_{ref}$, the concentration of marked molecules will stay constantly at $t_{ref}k_{-1}[C]$. Note that their decay rate v'_{-1} is not dependent of the concentrations of $A^\star$ or $B^\star$ in a steady state situation, but equals v_{-1} instead. Assuming an equilibrium by setting $v_1 = v'_{-1}$, we obtain $k_1[A][B] = k_{-1}[C]$. This system leads to the original equilibrium constant of $K = \frac{[C]}{[A][B]} = \frac{k_1}{k_{-1}}$ with the difference that the total concentrations are lower now. The new effective total concentration $[C'^0]$ which excludes the refractory molecules is now diminished by a multiplicative factor of $\frac{1}{1+t_{ref}k_{-1}}$.

$$K = \frac{[C]}{[A][B]} = \frac{k_1}{k_{-1}}, \qquad [C'^0] = [A] + [C] = [B] + [C] = \frac{C^0}{1 + t_{ref}k_{-1}}$$

Apparently, t_{ref} should be chosen so that the product $t_{ref}k_{-1} << 1$ to minimize the error.

References

[1] Gillespie DT: **A general method for numerically simulating the stochastic time evolution of coupled chemical reactions**. *J Comp Phys* 1976, **22**(4):403–434.

[2] Gillespie DT: **Exact Stochastic Simulation of Coupled Chemical Reactions**. *J Phys Chem* 1977, **81**(25):2340–2361.

[3] Takahashi K, Arjunan SNV, Tomita M: **Space in systems biology of signaling pathways–towards intracellular molecular crowding in silico.** *FEBS Lett* 2005, **579**(8):1783–1788.

[4] Berg OG, von Hippel PH: **Diffusion-Controlled Macromolecular Interactions**. *Annu Rev Biophys Biophys Chem* 1985, **14**:131–158.

[5] Schwartz R, Shor PW, Prevelige PE, Berger B: **Local rules simulation of the kinetics of virus capsid self-assembly.** *Biophys J* 1998, **75**(6):2626–2636.

[6] Andrews SS, Bray D: **Stochastic simulation of chemical reactions with spatial resolution and single molecule detail.** *Phys Biol* 2004, **1**(3-4):137–151.

[7] van Zon JS, Ten Wolde PR: **Green's-function reaction dynamics: A particle-based approach for simulating biochemical networks in time and space.** *J Chem Phys* 2005, **123**(23):128103–128103.

[8] Gillespie DT: **A diffusional bimolecular propensity function.** *J Chem Phys* 2009, **131**(16):164109, [http://dx.doi.org/10.1063/1.3253798].

[9] Solc K, Stockmayer W: **Kinetics of Diffusion-Controlled Reaction between Chemically Asymmetric Molecules. I. General Theory**. *J Chem Phys* 1971, **54**(7):2981.

[10] Shoup D, Lipari G, Szabo A: **Diffusion-controlled bimolecular reaction rates. The effect of rotational diffusion and orientation constraints.** *Biophys J* 1981, **36**(3):697–714, [http://dx.doi.org/10.1016/S0006-3495(81)84759-5].

[11] Konkoli Z, Johannesson H, Lee BP: **Fluctuation effects in steric reaction-diffusion systems**. *Phys. Rev. E* 1999, **59**(4):R3787–R3790.

[12] McNaught AD, Wilkinson A: *IUPAC. Compendium of Chemical Terminology: Iupac Recommendations (the "Gold Book").* Blackwell Scientific Publications, Oxford, 2nd edition 1997.
